# Olanzapine, but not haloperidol, exerts pronounced acute metabolic effects in the methylazoxymethanol rat model

**DOI:** 10.1111/cns.14565

**Published:** 2024-02-07

**Authors:** Katerina Horska, Silje Skrede, Jan Kucera, Gabriela Kuzminova, Pavel Suchy, Vincenzo Micale, Jana Ruda‐Kucerova

**Affiliations:** ^1^ Department of Pharmacology and Toxicology, Faculty of Pharmacy Masaryk University Brno Czech Republic; ^2^ Department of Clinical Science, Faculty of Medicine University of Bergen Bergen Norway; ^3^ Section of Clinical Pharmacology, Department of Medical Biochemistry and Pharmacology Haukeland University Hospital Bergen Norway; ^4^ RECETOX, Faculty of Science Masaryk University Brno Czech Republic; ^5^ Department of Physical Activities and Health, Faculty of Sports Studies Masaryk University Brno Czech Republic; ^6^ Department of Biomedical and Biotechnological Sciences, Section of Pharmacology University of Catania Catania Italy; ^7^ Department of Pharmacology, Faculty of Medicine Masaryk University Brno Czech Republic

**Keywords:** adipokine, antipsychotic, lipid profile, methylazoxymethanol, schizophrenia

## Abstract

**Aim:**

Widely used second‐generation antipsychotics are associated with adverse metabolic effects, contributing to increased cardiovascular mortality. To develop strategies to prevent or treat adverse metabolic effects, preclinical models have a clear role in uncovering underlying molecular mechanisms. However, with few exceptions, preclinical studies have been performed in healthy animals, neglecting the contribution of dysmetabolic features inherent to psychotic disorders.

**Methods:**

In this study, methylazoxymethanol acetate (MAM) was prenatally administered to pregnant Sprague–Dawley rats at gestational day 17 to induce a well‐validated neurodevelopmental model of schizophrenia mimicking its assumed pathogenesis with persistent phenotype. Against this background, the dysmetabolic effects of acute treatment with olanzapine and haloperidol were examined in female rats.

**Results:**

Prenatally MAM‐exposed animals exhibited several metabolic features, including lipid disturbances. Half of the MAM rats exposed to olanzapine had pronounced serum lipid profile alteration compared to non‐MAM controls, interpreted as a reflection of a delicate MAM‐induced metabolic balance disrupted by olanzapine. In accordance with the drugs' clinical metabolic profiles, olanzapine‐associated dysmetabolic effects were more pronounced than haloperidol‐associated dysmetabolic effects in non‐MAM rats and rats exposed to MAM.

**Conclusion:**

Our results demonstrate metabolic vulnerability in female prenatally MAM‐exposed rats, indicating that findings from healthy animals likely provide an underestimated impression of metabolic dysfunction associated with antipsychotics. In the context of metabolic disturbances, neurodevelopmental models possess a relevant background, and the search for adequate animal models should receive more attention within the field of experimental psychopharmacology.

## Graphical abstract

The figure shows the main methodological approaches and key results. FGF‐21, fibroblast growth factor 21; HAL, haloperidol; HDL, high‐density lipoproteins; LDL, low‐density lipoproteins; MAM, methylazoxymethanol model; OLA, olanzapine; TAG, triglycerides.

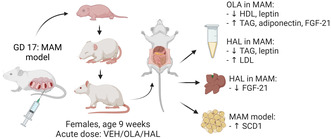

## 1 INTRODUCTION

During the past decades since the introduction of second‐generation antipsychotic agents, their potential for inducing adverse metabolic effects – diabetes, weight gain, and lipid disturbances – has become well‐known.^1^ Still, metabolic and cardiovascular adverse events remain a clinical challenge, with high prevalence and non‐satisfactory effects of lifestyle and pharmacological intervention alone or in combination.^2^, ^3^ Despite the extensive research interest, the understanding of the pharmacological and molecular mechanisms mediating the dysmetabolic effects of antipsychotics is insufficient and incomplete,^4^, ^5^ also due to the several confounding factors which are limiting the clinical studies, such as comorbidities and concomitant pharmacological treatment. In this context, preclinical models are irreplaceable research tools, allowing for controlled studies and direct examination of networks and tissues.^6^ This is an essential prerequisite for progress, as clinical studies in the vulnerable patient groups in question should rely on sound hypotheses.

The vast majority of preclinical studies concerning adverse metabolic effects of antipsychotics have been performed in healthy rodents.^7^, ^8^, ^9^, ^10^, ^11^, ^12^, ^13^ While such experiments can shed light on the pharmacological mechanisms triggering dysmetabolic features, there is strong evidence linking psychotic disorders and metabolic dysfunction even in untreated schizophrenic subjects.^14^, ^15^, ^16^, ^17^ The association between etiopathogenesis, underlying psychopathology, and adverse metabolic effects of antipsychotics is under‐investigated.^18^, ^19^, ^20^, ^21^, ^22^ In this regard, a major cause is that modeling of severe psychiatric disorders in animals is inherently challenging. A range of models relying on CNS lesions, pharmacological treatment, genetic deletions, or prenatal exposure have been introduced.^6^, ^23^ These models differ in theoretical background and the degree to which they meet validity criteria.^24^

The neurodevelopmental methylazoxymethanol acetate (MAM) model is based on prenatal administration of MAM (gestational day 17), which produces a phenotype characterized by persistent, functional, and neuropathological deficits mimicking schizophrenia in adult offspring.^21^, ^25^, ^26^ Therefore, the MAM model is an invaluable tool that reproduces the human condition in terms of construct, face, and predictive validity to investigate, in the present study, the significance of prenatal MAM exposure for the response to acute effects of treatment with the atypical antipsychotic olanzapine (OLA) and the typical antipsychotic haloperidol (HAL). These antipsychotics seem to affect metabolic status differentially. HAL has been shown to affect glucose, cholesterol, and TAG in humans, but it is a much less metabolically potent antipsychotic than OLA, an antipsychotic with a very high propensity to adverse metabolic effects.^1^, ^4^, ^27^

Clinically observed early weight gain is highly relevant and predicts long‐term weight gain during psychotropic treatment.^28^, ^29^ However, undesired metabolic features associated with antipsychotics can *precede* weight gain.^4^ Similarly, in rodents, a wide range of dysmetabolic effects occurring directly and not secondary to weight gain have been shown: both acute dysregulation of glucose homeostasis (hyperglycemia and decreased insulin sensitivity) and altered lipid metabolism.^4^, ^5^, ^30^, ^31^, ^32^ Adipose tissue dysfunction is a core feature of the pathophysiology of obesity and insulin resistance. At the same time, the adiponectin/leptin ratio is an emerging adipose tissue and metabolic function biomarker, which may serve as a predictor of cardiovascular risk.^33^, ^34^ Furthermore, in patients suffering from schizophrenia, the adiponectin/leptin ratio represents a potential marker of metabolic syndrome.^35^ Data on the acute effects of antipsychotics on endocrine hormones, for example, adipokines, incretins, and other metabolic regulators, for example, fibroblast growth factor 21 (FGF‐21) at the peripheral tissue level, are scarce.^4^, ^32^, ^36^, ^37^, ^38^, ^39^ Although there is evidence of hormonal dysregulation, its role remains unclear.^40^, ^41^

The rapid onset of feedback mechanisms may mask dysmetabolic alterations induced by antipsychotics during long‐term exposure.^9^, ^42^ In the present study, an acute setting allowed for the investigation of the direct effects of antipsychotics, independent of weight gain, an approach enabling mechanistic insight and suggesting potential therapeutic strategies. Thus, this study is focused on acute metabolic effects, including peripheral metabolic markers such as adipokine alterations and differences between the two drugs with specific dysmetabolic potential at 3 different consecutive timepoints over 24 h.

## 2 MATERIALS AND METHODS

Animal studies are reported in compliance with the ARRIVE guidelines.^43^ The study aimed to reveal metabolic disturbances induced by systemic OLA or HAL treatment in a well‐validated animal model of schizophrenia. Therefore, the use of experimental animals could not be avoided. Female Sprague–Dawley rats were used for consistency with previous literature. This strain and sex are optimal for developing the OLA‐induced phenotype, such as increased body weight and food intake.^8^, ^9^, ^36^

### 2.1 Animals

Time‐mated female albino Sprague–Dawley rats were purchased from Charles River (Germany) at gestational day (GD) 13 and housed individually. They were randomly assigned to saline‐treated group (control group – CTR) or methylazoxymethanol acetate‐treated group (MAM). The MAM model was induced as previously described and validated.^44^, ^45^, ^46^, ^47^, ^48^, ^49^, ^50^ Briefly, MAM was dissolved in saline and intraperitoneally administered at a dose of 22 mg/kg in 1 mL/kg volume on GD 17. The offspring were weaned on a postnatal day (PND) 22 and housed in groups of 2–3. The study was performed in 24 CTR and 30 MAM female offspring at 9 weeks of age. Selection of the female subjects adheres to the standard approach in this field and our previous data.^8^, ^10^, ^11^, ^36^, ^51^ The experimental groups were as follows: CTR‐VEH (*n* = 8), CTR‐OLA (*n* = 8), CTR‐HAL (*n* = 8), MAM‐VEH (*n* = 10), MAM‐OLA (*n* = 10), and MAM‐HAL (*n* = 10).

Environmental conditions during the whole study were constant: relative humidity 50–60%, temperature 23 ± 1°C, and a normal 12‐h light–dark cycle (6 a.m. to 6 p.m. light). Standard rodent chow and water were available ad libitum. All procedures were performed in accordance with EU Directive No. 2010/63/EU and approved by the Animal Care Committee of the Faculty of Medicine, Masaryk University, Czech Republic, and the Czech Governmental Animal Care Committee, in compliance with Czech Animal Protection Act No. 246/1992.

### 2.2 Drugs and treatments

**FIGURE 1:**
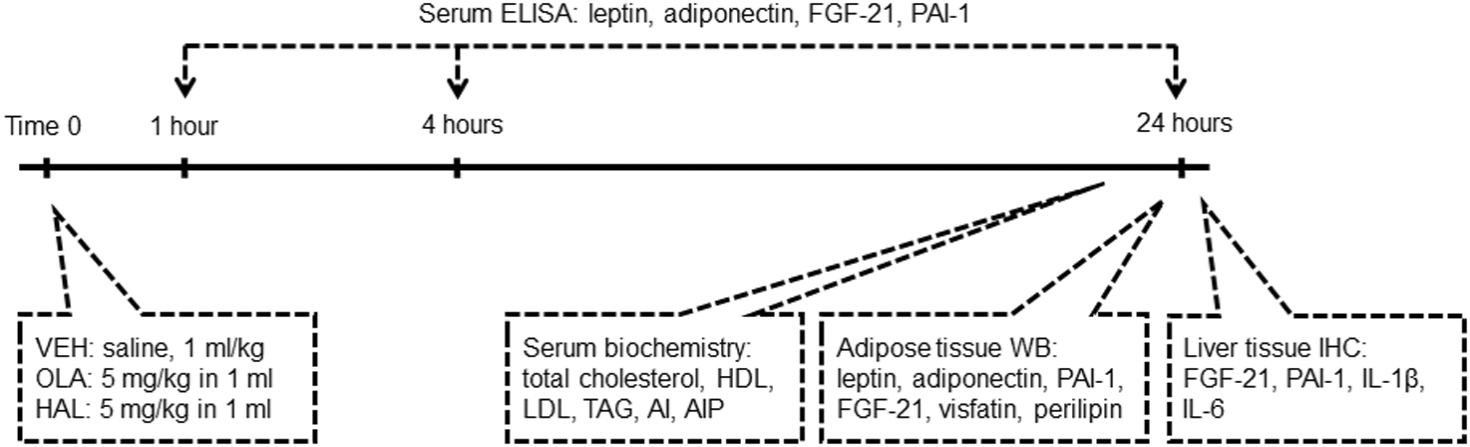
Schematic diagram of the study design. The timeline indicates the design of the study, blood sampling, and all analyses performed at specific timepoints.

Methylazoxymethanol acetate (MAM; Midwest Research Institute, Kansas City, USA) was dissolved in saline and administered intraperitoneally at a 22 mg/kg dose in 1 mL/kg volume on GD 17. Saline was injected to the control group as a vehicle (CTR). The timeline and design of the study are depicted in Figure 1. Both antipsychotics were acutely administered as intramuscular injections designed for human use at 5 mg/kg in 1 mL, and saline was used as vehicle control (VEH): olanzapine (OLA): Zyprexa® (Eli Lilly Nederland BV, Utrecht, The Netherlands) and haloperidol (HAL): Haloperidol® (Gedeon Richter Plc., Budapest, Hungary). The doses of both drugs were chosen based on calculations and extrapolations, considering pharmacokinetic parameters, especially the much shorter half‐life of antipsychotics in rodents compared with that in humans. Doses with regard to the route of administration of these antipsychotics were selected based on plasma concentrations and in vivo D_2_‐receptor occupancies in rats^8^, ^52^ and clinical dosing schemes.

### 2.3 Sample collection

Approximately 1 mL of blood was collected under short isoflurane (AERRANE®, Baxter S.A., Belgium) anesthesia by retro‐orbital puncture one and 4 h after drug administration.^53^ The intraperitoneal injection of saline supplied the same amount of liquid. Twenty‐four hours after drug administration, all rats were sacrificed by decapitation under isoflurane anesthesia to collect blood (for serum). Dissection was performed after decapitation by wide laparotomy, with sampling from the liver and white abdominal tissues. The samples were frozen at −70°C until laboratory analysis.

### 2.4 Biochemical assays

Leptin, adiponectin, FGF‐21, and PAI‐1 were assessed by an immunochemical method (ELISA) using commercial sets (BioVendor®). The quantification limits of individual analyses were as follows: leptin – 100 pg/mL; adiponectin – 200 pg/mL; FGF‐21 – 40 pg/mL; and PAI‐1 – 298 pg/mL. The quantification limit was used in the case of analysis results under the quantification limit. This was the case of 14 values in leptin analysis, 20 values in FGF‐21 analysis, and 12 values in PAI‐1 analysis (no adiponectin values were under the limit). The leptin/adiponectin ratio was calculated as previously suggested.^35^ All analyses were performed using serum samples withdrawn 1 and 4 h after drug administration and in serum samples obtained during dissection 24 h after drug dosing (three timepoints).

Lipid parameters were determined in serum samples obtained 24 h after drug dosing by enzymatic, colorimetric (total cholesterol, HDL), or photometric (LDL, TAG) method (Roche/Hitachi COBAS C®) by Lab Med s.r.o., Brno, Czech Republic. Furthermore, the atherogenic index (AI) was calculated as a ratio of total cholesterol/HDL, and the atherogenic index of plasma (AIP) was calculated as the log(TAG/HDL). The reason for analyzing only the last timepoint was the small amount of blood (and subsequently serum), which was possible to collect in the early timepoints together with a relatively high demand of sample volume for this analysis (approx. 50 μL per assayed analyte).

### 2.5 Western blot analysis of white adipose tissue

Sample preparation and Western blot analysis were performed by a standard procedure as presented previously.^54^ Briefly, approximately 5 mm^3^ of adipose tissue was minced using sterile forceps and a scalpel. Fragments were collected to a sterile Eppendorf tube and lysed in 300 μL of SDS lysis buffer (1% SDS, 10% glycerol, 100 mM Tris‐Cl pH 7.4). Samples were vortexed vigorously for 1 min, followed by centrifugation at 14,000 rpm for 1 min. This step was repeated three times. According to the manufacturer's instructions, total protein concentration was determined using DC protein assay (Bio‐Rad, USA), and all samples were equalized for protein content. Following the addition of 2‐mercaptoethanol (1%) and bromophenol blue (0.01%), the samples were boiled for 5 min at 95°C. An equal amount of protein (30 μg) was separated by SDS‐PAGE. Proteins were transferred to a polyvinylidene difluoride membrane (Millipore, USA). Western blot analysis was performed using standard protocols with the following primary antibodies: Adiponectin (C45B10) Rabbit mAb #2789, FABP4 (fatty acid‐binding protein 4) Antibody #2120, Perilipin‐1 (D1D8) XP® Rabbit mAb #9349, Stearoyl‐CoA desaturase‐1 (SCD1, C12H5) Rabbit mAb #2794, Vinculin (E1E9V) XP® Rabbit mAb #13901 (all Cell Signaling Technology, USA), Recombinant Anti‐Visfatin antibody [EPR21984] (ab236873), Recombinant Anti‐FGF21 antibody [EPR8314 (2)] (ab171941), Recombinant rabbit anti‐PAI‐1 (ab66705) (all Abcam, UK), Leptin Polyclonal Antibody #PA1‐051 (Invitrogen, USA), Secondary Anti‐rabbit IgG, HRP‐linked Antibody #7074, and Anti‐mouse IgG, HRP‐linked Antibody #7076 (both Cell Signaling Technology, USA) were used in conjunction with Immobilon Western Chemiluminescent HRP Substrate (Millipore, USA) to visualize immunoreactive bands.

Densitometry analysis was performed using ImageJ software (NIH, USA), and relative protein expression was calculated after normalization against vinculin. The data are presented as a fold change of the CTR‐VEH group, that is, CTR‐VEH group mean levels = 1 ± SD. Samples were distributed in 4 gels. Each one contained 3 samples of the CTR‐VEH group and 2 samples of the other 5 experimental groups, allowing for analysis of *n* = 8 samples in the CTR‐VEH group and *n* = 6 in all other groups.

### 2.6 Histopathological assessment and immunohistochemistry of the liver tissue

Liver samples were thawed, fixed in 10% buffered paraformaldehyde (Bamed, Czech Republic), and embedded in paraffin. Then, the samples were cut using a sliding microtome (Leica SM 2000R, Leica Biosystems, USA) with a slice thickness of 1.5 μm and stained with hematoxylin–eosin (hematoxylin – Bamed, Czech Republic; eosin – Merck, USA). An experienced blinded observer performed a histopathological assessment. The liver tissue was examined particularly for the presence of inflammatory signs (infiltrated portobilium or inflammatory infiltrate in liver sinusoids), steatosis (presence and extent), and fibrosis (particularly increase of fibrous tissue in portobilium as a result of the reparatory process). The samples were manually scored to 3 degrees of intensity (0 – normal finding; 1 – subtle changes; and 2 – distinct pathological changes).

Immunohistochemical (IHC) staining was performed on 1.5‐μm tissue sections applied to positively charged slides. Antibodies against FGF‐21 (Recombinant Anti‐FGF21 rabbit antibody, EPR8314 (2), ab171941, Abcam, UK; dilution 1:250), PAI‐1 (Anti‐PAI‐1 rabbit antibody, ab66705, Abcam, UK; dilution 1:200), IL‐1beta (Anti‐IL‐1 beta rabbit antibody, ab9722, Abcam, UK; dilution 1:500), and IL‐6 (Anti‐IL‐6 mouse antibody, ab9324, Abcam, UK; dilution 1:500) were used. The prepared specimens were stained using Ventana BenchMark® automated staining (BenchMark ULTRA – Ventana Medical Systems, Roche, Switzerland) according to the manufacturer's instructions.

A blinded observer evaluated all immunostained slides using a light microscope at 400x magnification. The intensity of staining and the percentage of positive cells were assessed at least in five high‐power fields of the tissue section. In the majority of samples, the whole area was assessed. The expression of IHC markers was classified into four levels: no expression (0); light expression, less than 25% of positive cells (1); medium expression, 25%–50% of positive cells (2); and very significant expression, more than 50% of positive cells (3).

### 2.7 Statistical data analysis

Primary data were summarized using the arithmetic mean and standard deviation (±SD). All analyses were calculated using IBM SPSS Statistics 28.0.0.0 (190). A value of *p* < 0.05 was set as the boundary of statistical significance in all applied tests. The data were tested for normality by the Shapiro–Wilk test and analyzed by a parametric or non‐parametric method as appropriate. The study design features two factors – the MAM model (CTR‐MAM) and treatment (VEH‐OLA‐HAL). For body weight and serum lipid analysis, a two‐way (2 W) ANOVA was performed with Tukey's post‐hoc test when significant differences were detected in the treatment factor or model*treatment interaction. To analyze a repeated sampling of serum adipokine levels (ELISA), a repeated measures approach was selected to test the effect of the MAM model and treatment in three consecutive measurements. The mixed‐effects model was used with Sidak's post‐hoc test when appropriate. For Western blot analysis of adipose tissue and immunohistochemical staining of liver slices, a non‐parametric approach was used – the Kruskal–Wallis test. For the subgroup analysis of selected biochemical variables (CHOL, HDL, LDL, TAG, AI, AIP, leptin, adiponectin, and L/A ratio) in CTR‐OLA and MAM‐OLA rats, where responders and non‐responders were found in the MAM‐OLA group, the Kruskal–Wallis test was used due to a low number of subjects (*n* = 4–5).

## 3 RESULTS

### 3.1 Body weight

All rats were weighed at the time of drug administration. The average BW in the CTR rats was 227 g (±SD 14.47) and in the MAM rats was 192 g (±SD 12.82). The 2 W ANOVA indicated a significant effect of the MAM model (*F*_1,46_ = 79.889, *p* < 0.001), with significantly lower BW in the MAM rats. No effect of the assignment to a treatment group was observed, and the data had equal variability (data not shown).

### 3.2 Adipokine levels 1, 4, and 24 h after drug administration

**FIGURE 2:**
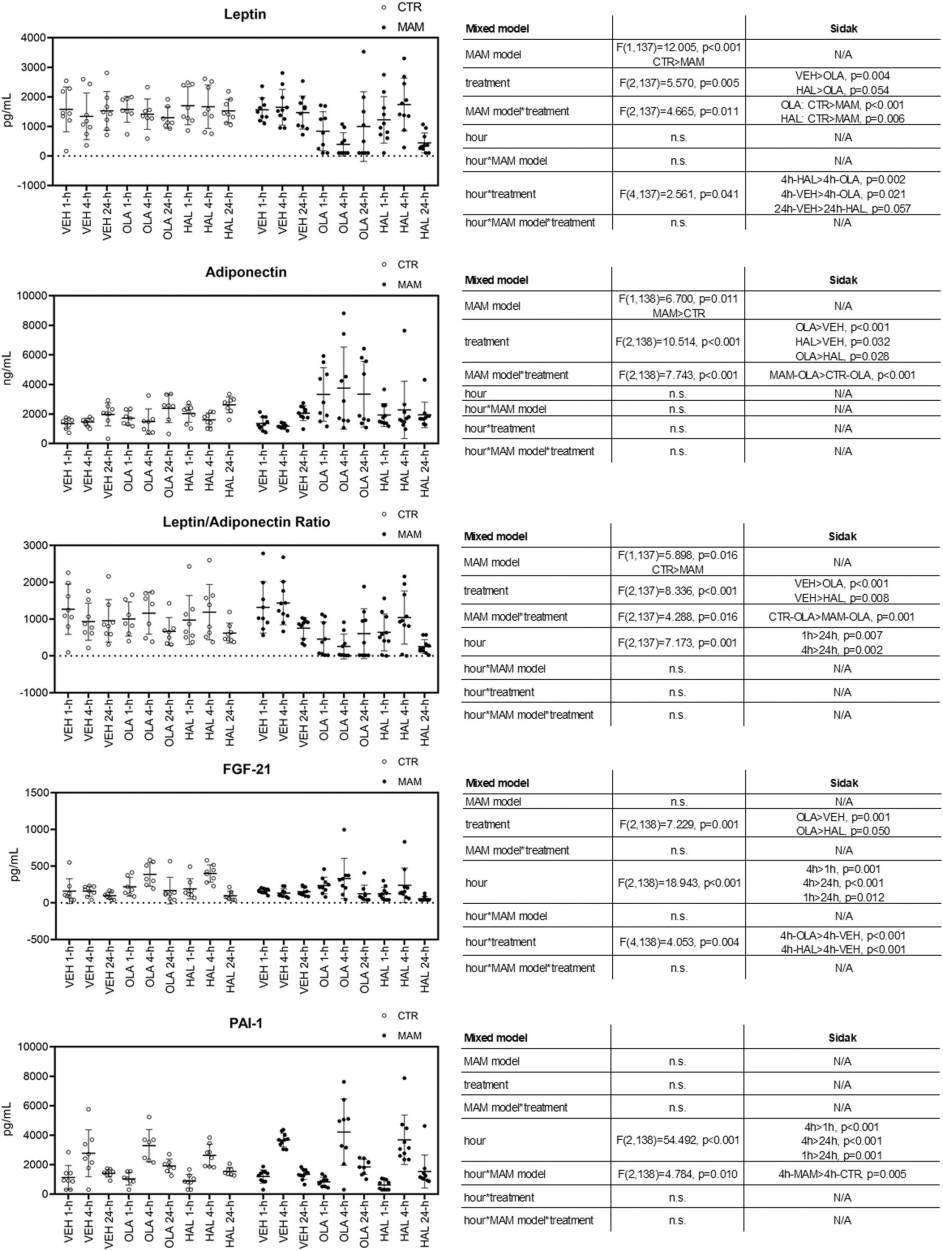
Adipokine levels at 1, 4, and 24 h after treatment. The data are presented as means ±SD and individual data points. The tables show the results of the mixed‐effects model and Sidak's post‐hoc test where applicable.

As apparent from Figure 2, with *p* values presented in the right‐hand panel, in the serum ELISA assessment of adipokines, the mixed model showed a significant overall effect of the MAM model on leptin (lower than in CTR rats), adiponectin (higher than in CTR rats), and their ratio (lower than in CTR rats). There were numerous significant effects of antipsychotic treatment. Particularly, OLA decreased leptin and leptin/adiponectin ratio and increased adiponectin and FGF‐21. HAL increased adiponectin and decreased the leptin/adiponectin ratio. In some cases, these effects were different in the MAM and CTR rats. Specifically, OLA‐induced decrease of leptin and leptin/adiponectin and increase of adiponectin were present in MAM rats only. HAL‐induced effect on leptin had the same pattern, showing a decrease after acute dosing. The mixed model did not reveal a significant effect of the main factors (MAM model or treatment) in PAI‐1.

The time‐dependent analysis showed differential temporal patterns in leptin, FGF‐21, and PAI‐1 levels. Leptin level was significantly lower 4 h after OLA administration compared to the same timepoint in VEH‐treated rats. HAL showed a similar effect 24 h after dosing (trend toward significance, *p* = 0.057). FGF‐21 level was significantly higher 4 h after both OLA and HAL administration compared to the same timepoint in VEH‐treated rats. The analysis of PAI‐1 data showed a non‐specific increase in its levels 4 h after treatment, particularly in the MAM rats. This includes not only the antipsychotics but also VEH treatment, suggesting a non‐specific effect exerted by the manipulation rather than pharmacological treatment.

### 3.3 Serum lipid profile 24 h after drug administration

**FIGURE 3:**
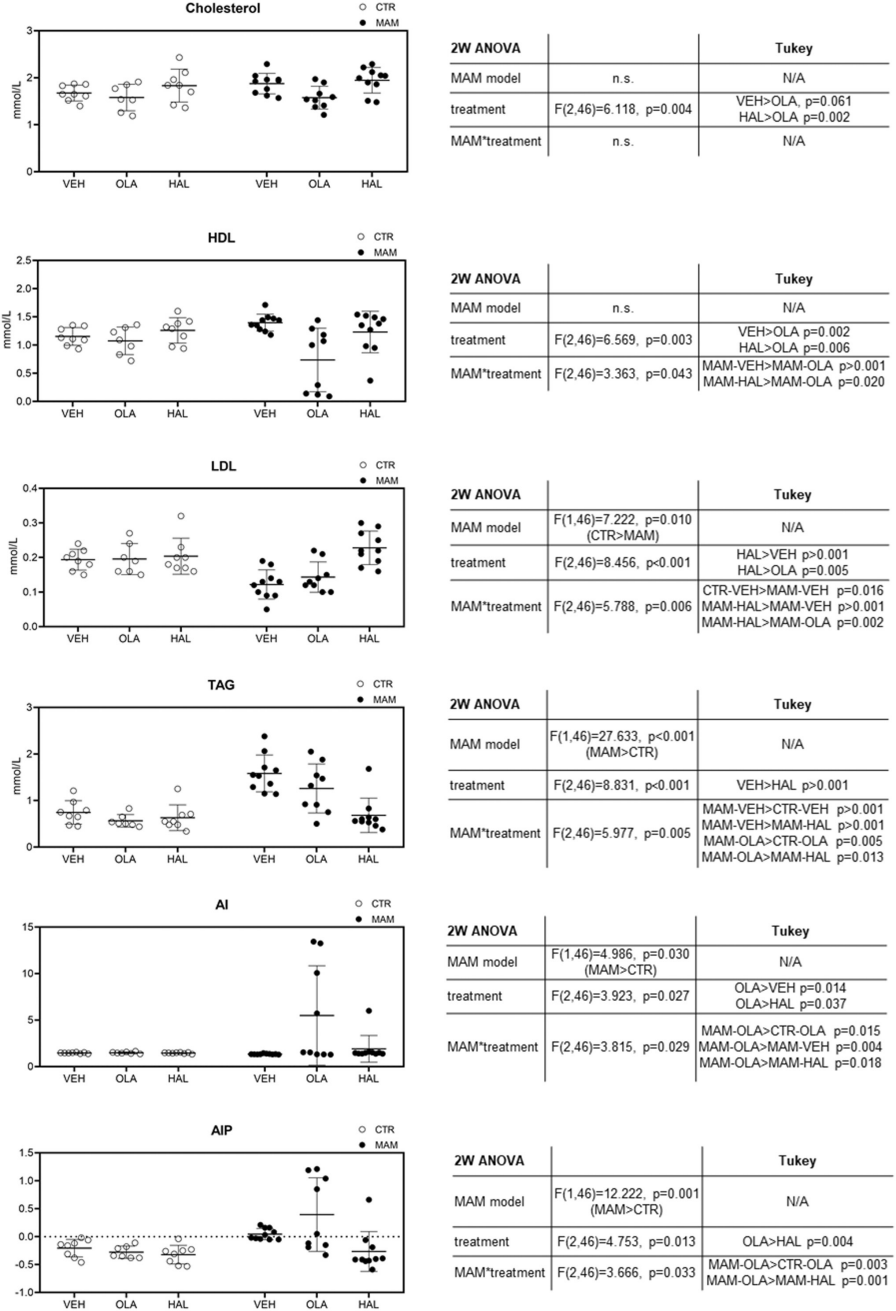
Serum lipids at 24 h after treatment. The data are presented as means ±SD and individual data points. The tables show the 2 W ANOVA and Tukey's post‐hoc test results where applicable.

The complete results of the statistical analysis are presented in Figure 3, together with a graphical representation of the data. In the serum lipid profile, the 2 W ANOVA detected a significant effect of the MAM model in LDL (lower than in CTR rats), TAG, AI, and AIP (higher than in CTR rats). The effect of antipsychotics was found to be significant for all assessed variables. Specifically, OLA decreased the levels of CHOL and HDL and increased AI compared to VEH and/or HAL treatments. It also increased AIP in comparison with HAL. HAL increased LDL and decreased TAG (vs VEH treatment). Notably, many of these effects were present in the MAM rats only (results of MAM model*treatment interaction). This applies particularly to the OLA‐induced decrease of HDL and increase of AI, which were present only in MAM rats. Similarly, HAL induced an increase in LDL and a decrease in TAG in MAM rats (MAM‐HAL vs. CTR‐HAL). Furthermore, OLA increased TAG, AI, and AIP in MAM rats more than in the CTR cohort.

#### 3.3.1 Two metabolic subgroups among MAM‐OLA rats

The data obtained in this study were all found to be normally distributed (except for the ordinal data from the IHC staining). However, when carefully examined, the raw data showed that based on their metabolic response, rats in the MAM‐OLA group fell into either of two subgroups: In 4 rats, HDL cholesterol and leptin levels were markedly lower, while TAG and adiponectin levels were higher than in the remaining 5 rats. On the other hand, LDL and total cholesterol values were equal in all rats (Table 1). A sub‐analysis distinguishing between responders and non‐responders was performed based on these observations. The MAM‐OLA rats were deemed responders when the total CHOL minus HDL minus LDL level was equal to or higher than 1 mmol/L (notably, mean values in the CTR‐OLA group and non‐responders in the MAM‐OLA rats were 0.31 mmol/L in both groups). The Kruskal–Wallis test identified multiple significant differences between the MAM‐OLA‐R (responder) group vs. CTR‐OLA and MAM‐OLA‐NR (non‐responder) groups. The same pattern was observed in one MAM‐HAL rat but not in any other rats in the experiment. The whole dataset is included in the Supplementary Materials.

**TABLE 1:** OLA‐induced metabolic response in MAM rats.

	CTR‐OLA	MAM‐OLA‐NR	MAM‐OLA‐R	Kruskal–Wallis test	
CHOL	1.58 ± 0.26	1.66 ± 0.23	1.47 ± 0.17	*H* (2)=0.72, *p* = 0.699	n.s.
HDL	1.07 ± 0.23	1.20 ± 0.16	0.16 ± 0.08	*H* (2)=8.78, *p* = 0.012	MAM‐OLA‐R<CTR‐OLA, *p* = 0.041 MAM‐OLA‐R<MAM‐OLA‐NR, *p* = 0.016
LDL	0.20 ± 0.04	0.16 ± 0.05	0.12 ± 0.01	*H* (2)=6.47, *p* = 0.039	MAM‐OLA‐R<CTR‐OLA, *p* = 0.036
TAG	0.56 ± 0.12	0.88 ± 0.27	1.74 ± 0.24	*H* (2)=10.66, *p* = 0.005	MAM‐OLA‐R>CTR‐OLA, *p* = 0.003
AI	1.49 ± 0.09	1.39 ± 0.11	10.62 ± 3.13	*H* (2)=9.45, *p* = 0.009	MAM‐OLA‐R>MAM‐OLA‐NR, *p* = 0.008
AIP	−0.28 ± 0.10	−0.15 ± 0.12	1.07 ± 0.15	*H* (2)=10.53, *p* = 0.006	MAM‐OLA‐R>CTR‐OLA, *p* = 0.004
Leptin	1294.49 ± 338.29	1710.96 ± 1035.87	100.00 ± 0.00	*H* (2)=8.79, *p* = 0.012	MAM‐OLA‐R<CTR‐OLA, *p* = 0.036 MAM‐OLA‐R<MAM‐OLA‐NR, *p* = 0.019
Adiponectin	2376.72 ± 887.39	1511.43 ± 270.84	5626.36 ± 616.96	*H* (2)=10.14, *p* = 0.006	MAM‐OLA‐R>MAM‐OLA‐NR, *p* = 0.005
L/A ratio	0.67 ± 0.35	1.07 ± 0.49	0.02 ± 0.00	*H* (2)=9.98, *p* = 0.007	MAM‐OLA‐R<MAM‐OLA‐NR, *p* = 0.006

*Note*: The data are presented as means ±SD. Statistical results of the Kruskal–Wallis ANOVA are presented.

### 3.4 Western blot analysis of the white adipose tissue

**FIGURE 4:**
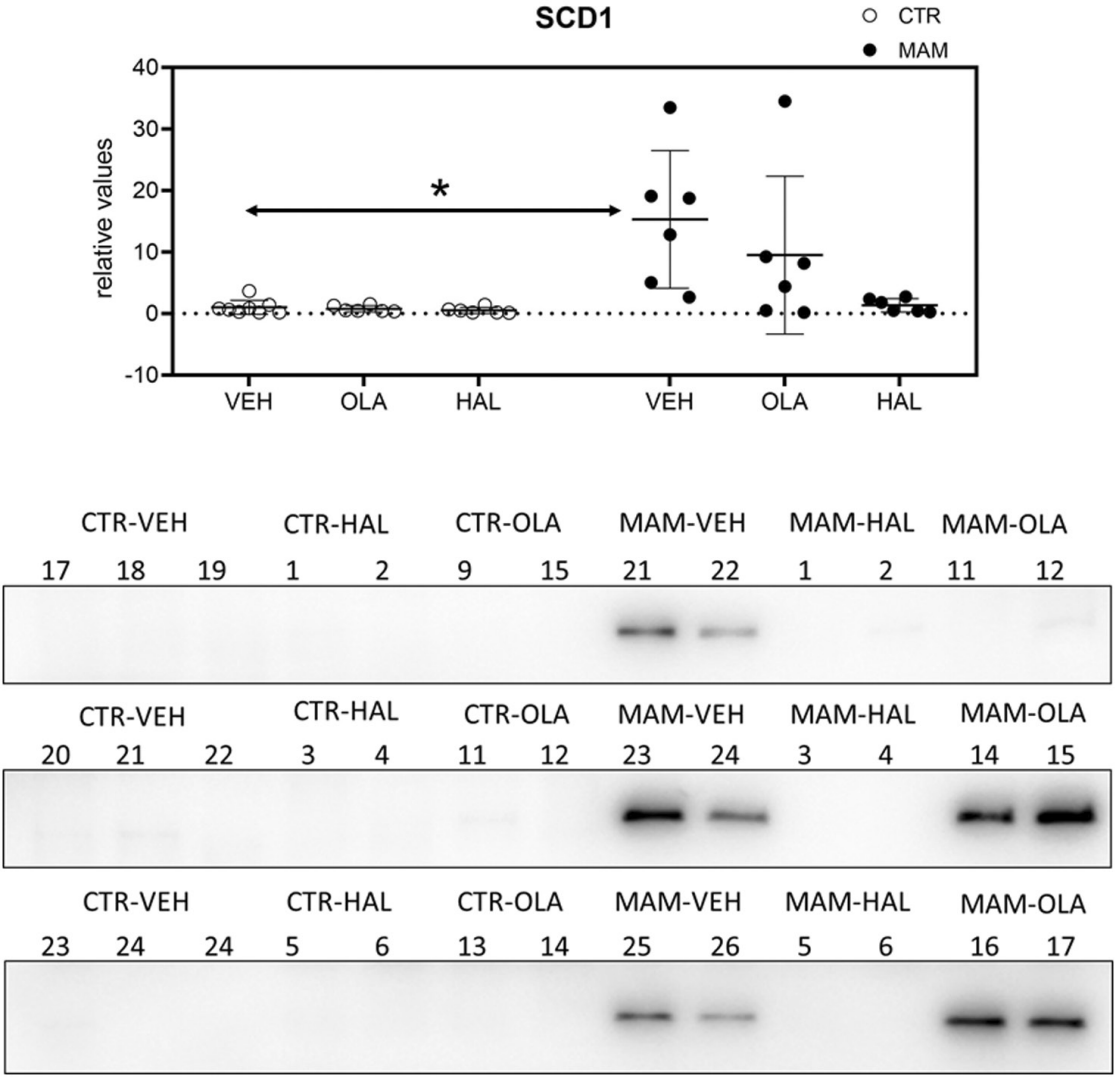
Western blot analysis of SCD1 (37 kDa) in adipose tissue 24 h after treatment. The data are presented as means ±SD and individual data points. The statistical result of the Kruskal–Wallis test is depicted in the graph, *p < 0.05. Note that due to the organization of samples on the gel and the consistency of the graphs in this paper, the experimental groups are organized differently in the graph and the photograph of the gels below it.

A non‐parametric approach was used for the statistical analysis of Western blot data – the Kruskal–Wallis test. The only protein showing significant differences among the experimental groups was SCD1: *H* (5)=16.537, *p* = 0.006 (Figure 4). A two‐tailed test for multiple comparisons revealed a higher amount of the protein in the adipose tissue of MAM‐VEH animals compared to the CTR‐VEH group (*p* = 0.042) but no significant effect of antipsychotics. The Kruskal–Wallis test did not indicate significant differences in perilipin (*H* (5)=6.622, *p* = 0.250), PAI‐1 (*H* (5)=3.074, *p* = 0.689), adiponectin (*H* (5)=2.295, *p* = 0.807), leptin (*H* (5)=3.397, *p* = 0.639), visfatin (*H* (5)=5.222, *p* = 0.389), and FGF‐21 (*H* (5)=5.733, *p* = 0.333). Photographs of the gels are included in Supplementary Materials.

### 3.5 Histology and immunohistochemical staining of the liver tissue

The liver histological examination was performed to determine pathological changes induced by prenatal MAM exposure. No difference in inflammatory signs, steatosis, or fibrosis was detected as an index of pathological changes between the MAM and CTR groups. Given the entirely physiological state of the tissue, the photographs are not presented.

**FIGURE 5:**
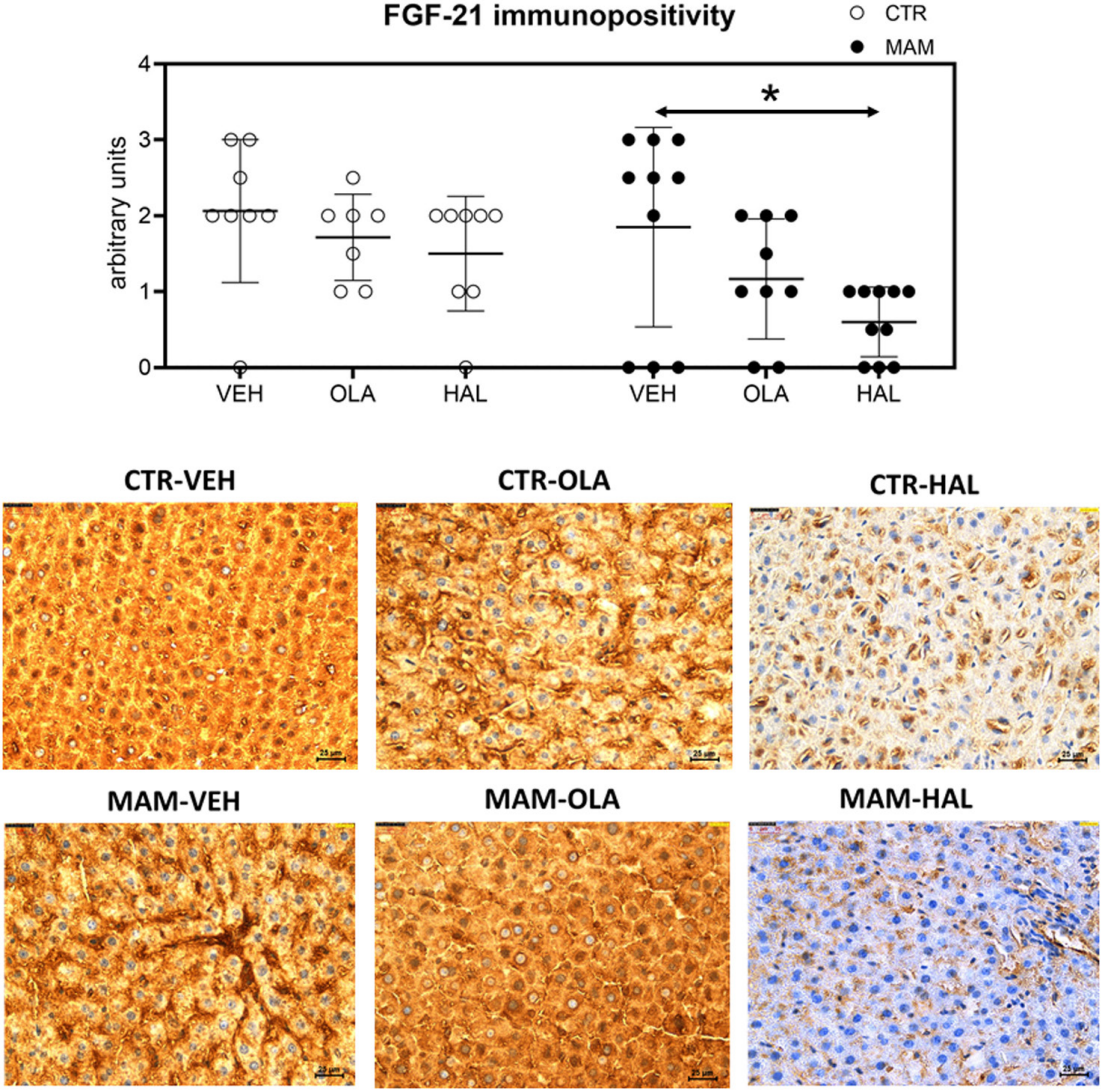
Immunohistochemical staining of liver 24 h after treatment. The data are presented as means ±SD and individual data points. Statistical results of the Kruskal–Wallis ANOVA are depicted in the graph. The photographs show representative samples of each group to illustrate the results obtained in the statistical analysis, *p < 0.05. Photograph magnification is 400×, and scale bars are in the right down corner (25 μm). The brown color shows immunopositivity for FGF‐21; blue is the background signal.

For the immunohistochemical staining, the Kruskal–Wallis analysis revealed a significant FGF‐21 immunopositivity in the liver tissue: *H* (5)=15.144, *p* = 0.010. A two‐tailed test for multiple comparisons identified a significantly higher FGF‐21 immunopositivity in MAM‐VEH animals compared to MAM‐HAL (*p* = 0.039). The data are shown in Figure 5. No differences were detected in the levels of PAI‐1 in the liver tissue as the test showed a significant result (*H* (5)=12.696, *p* = 0.026). However, the test for multiple comparisons did not reveal any significantly different experimental groups. Immunohistochemical staining of IL‐1beta and IL‐6 in the liver tissue revealed too low, barely detectable expression levels in all groups.

## 4 DISCUSSION

In this study, the acute impact of the metabolically potent antipsychotic OLA and the less metabolically potent drug HAL was compared for a range of metabolic parameters in the MAM neurodevelopmental rat model. The aim of using the MAM model, like other neurodevelopmental models of psychotic disorders, is to produce a phenotype as closely related to first‐episode psychosis (FEP) as possible. Indeed, FEP is associated with weight gain and lipid‐ and glucose‐related disturbances present before the initiation of antipsychotic treatment. Altered glucose homeostasis, specifically insulin regulation, and impaired glucose tolerance, as well as derangements in lipid metabolism, are consistently reported in FEP populations,^55^ manifesting as hypertriglyceridemia, insulin resistance, and reduced total and LDL cholesterol levels.^56^ In humans, recent meta‐analyses reported increased rates of metabolic syndrome or at least one metabolic syndrome criterion even in drug‐naïve FEP, suggesting underestimated cardiovascular risk in this population. With data underlining the notion that altered metabolic parameters are not exclusively associated with antipsychotic exposure, the authors also stressed the need to focus on metabolic risk predictors specific to the FEP population.^57^, ^58^ Nevertheless, antipsychotic treatment contributes significantly to metabolic derangements in patients, with associated cardiovascular disease risk^1^, ^14^ demonstrated even in the absence of psychiatric illness and also preclinically.^59^

Concerning antipsychotic‐naïve metabolic phenotype in schizophrenia‐like animal models, only a limited number of studies reported metabolic parameters.^18^, ^36^ In our previous studies, the MAM model was associated with serum lipid profile alterations in both sexes; total, HDL, and LDL cholesterol levels were increased.^36^ In another neurodevelopmental model induced by prenatal administration of the polyinosinic:polycytidylic acid (poly I:C), disturbance of lipid metabolism was also observed.^18^ Our data in MAM and poly I:C models consistently indicate intrinsic lipid alterations even in drug‐naïve animals.

Unlike in our previous study, where prenatal MAM exposure did not affect body weight, MAM exposure resulted in lowered body weight in the present cohort, demonstrating the vulnerability induced by MAM exposure.^36^ However, the alterations in lipid parameters in MAM‐treated animals – LDL, TAG, AI, AIP, serum adiponectin, and leptin serum levels and their ratio, as well as protein levels of the fatty acid desaturase *Scd1* in white adipose tissue of female rats – are not likely to result solely from reduced body weight. To our knowledge, this is one of the most extensive studies of lipid profile characterization, adipokine, or other peripheral mediator levels in the MAM model.^36^ Thus, lipid alterations could represent a trait of neurodevelopmental models reflecting the association of dyslipidemia in antipsychotic naïve FEP patients^60^ in accordance with the assumed link between lipid homeostasis and schizophrenia^61^ with potential genetic overlap.^62^

With regard to the effects of antipsychotics, OLA had significantly more pronounced effects in female MAM rats than in non‐MAM (CTR) rats for several readouts. Moreover, in the MAM‐OLA group, roughly 50% of the animals had a pronounced response to OLA, with serum lipid and adipokine levels significantly deviating from those found in the CTR‐OLA group, that is, non‐MAM animals treated with OLA. HDL and LDL cholesterol parameters, leptin and leptin/adiponectin ratio were significantly reduced, while TAG, AI, AIP, and adiponectin were massively increased in the MAM‐OLA responder group compared to the CTR‐OLA group. These effects were in line with previous acute studies in healthy animals,^27^, ^32^, ^42^ supporting the current understanding of weight‐independent dysregulation of lipid metabolism.^5^, ^63^ Indeed, preclinical evidence for direct effects of antipsychotics, with consistent findings of increased TAG, possibly reflect a compensatory response to inhibited cholesterol biosynthesis.^5^, ^30^, ^36^, ^63^ The molecular mechanisms underlying dyslipidemia are still incompletely elucidated; nevertheless, the alterations lead to decreased HDL and increased very low‐density lipoprotein and TAG.^5^

We carefully considered and eliminated several potential confounders for the subgroup phenomenon (differences in feeding, body weight, litter effects, housing specifics, infection or sickness, and preanalytical and analytical issues). According to our interpretation of the subgroup formation, MAM treatment leads to a delicately balanced metabolic state, where OLA exposure triggers pronounced acute metabolic responses in vulnerable individuals. Our investigation did not reveal the cause and mediators of this state but should be subject to further scrutiny. In terms of translational value, although the MAM model has obvious shortcomings, the sharp distinction between responders and non‐responders to OLA is interesting in identifying patients vulnerable to metabolic side effects through biomarker measurements.^64^

Regarding endocrine markers in serum, OLA was associated with decreased leptin and leptin/adiponectin ratio and increased adiponectin. Moreover, a robust transient increase in serum FGF‐21 was observed. For these markers, HAL, despite being considered metabolically less risky, increased adiponectin and decreased leptin/adiponectin ratio. HAL administration affected serum markers in the MAM model: It reduced serum leptin in MAM rats and decreased hepatic FGF‐21 immunopositivity. It induced a transient serum increase of FGF‐21 in both CTR and MAM cohorts. This study shows antipsychotic‐associated acute alterations in the leptin/adiponectin ratio for the first time, together with an increase in FGF‐21.

Data on FGF‐21 in schizophrenia are limited. One study reported elevated levels even in first‐onset patients with schizophrenia.^65^ Although the FGF system is considered a potential target in schizophrenia,^66^ there are no data on the effect of antipsychotics on FGF‐21.^67^ FGF‐21, as a mediator of energy homeostasis, represents a target for emerging new potentially beneficial therapies for obesity, dyslipidemia, and glucose dysregulation^68^, ^69^, ^70^ and possibly in the development of strategies in prevention or treatment of adverse metabolic effects of antipsychotics.

The present study was conducted in female rats. Recently reviewed clinical and preclinical data regarding sex differences in antipsychotic‐associated metabolic disturbances report females at higher risk of weight gain and diagnosis of metabolic syndrome. However, accumulation of adipose tissue depots and glucose homeostasis dysregulation also occur in male rats and are considered independent of weight changes.^27^ Resonating with this, while male rats do develop features such as adiposity, lipogenic activation, and adverse lipid profile in the absence of weight gain, female rats are more susceptible to antipsychotic‐induced weight gain and related dysmetabolic features.^8^, ^71^ In our previous experiments, sex‐dependent alterations in lipid profile were found; the female sex was associated with higher total cholesterol and HDL, whereas LDL, AI, AIP, and CRP were higher in male rats in both control and MAM cohorts.^36^ In summary, female rats are considered more relevant for modeling antipsychotic‐associated weight gain than male rats, concerning both face and construct value. Therefore, the female model was utilized in the present experiment.

## 5 CONCLUSIONS

In summary, our results demonstrate that prenatal MAM exposure led to a metabolic vulnerability in female rats, with pronounced effects of OLA in approximately 50% of the MAM cohort and less pronounced HAL effects in accordance with the respective drugs' degree of clinical impact on metabolism.^1^, ^4^ However, a central issue affecting the validity of the MAM model is whether MAM treatment yields metabolic effects comparable to those found in schizophrenia/psychosis. Determining a clear pattern of the metabolic profile of these psychiatric disorders is complicated, as numerous studies did not take antipsychotic medication unequivocally into account, as many patients in the studies were not treatment naïve. Moreover, translational aspects regarding serum lipid parameters prevent direct comparison between absolute changes in serum lipid species between rodents and humans.^72^ Nevertheless, the noticeable impact of a neurodevelopmental model on baseline lipid metabolism and the metabolic response to OLA underlines that findings from healthy rats are likely to provide an underestimated impression of metabolic dysfunction. Neurodevelopmental models possess the most relevant background in the context of metabolic disturbances. The search for adequate animal models should receive more attention within the field of experimental psychopharmacology.

## AUTHOR CONTRIBUTIONS

KH prepared the design and protocol of the study and contributed to writing the manuscript. SS initiated the interpretation of the results and contributed to writing the manuscript. JK processed the samples and conducted the laboratory analyses (Western blot). GK processed the samples and conducted laboratory analyses (histology and immunohistochemistry). PS coordinated the laboratory analyses. VM developed the MAM model and prepared the animals for the study. JRK planned the experiments, conducted the in vivo part of the study, collected all data, performed the statistical analysis, prepared figures and tables, and contributed to writing the manuscript. All authors revised and edited the final manuscript.

## FUNDING INFORMATION

This study was performed at Masaryk University as part of the projects “Preclinical and clinical research in pharmacokinetics, neuropsychopharmacology and personalized pharmacotherapy in oncology” number MUNI/A/1342/2022 and “Preclinical development of therapeutic approaches in reduction of adverse metabolic effects of atypical antipsychotics” number MUNI/A/1372/2020 with the support of the Specific University Research Grant, as provided by the Ministry of Education, Youth and Sports of the Czech Republic in the years 2021 and 2023. This work was also supported by the SoMoPro II Programme (project No. 3SGA5789). JK thanks the RECETOX Research Infrastructure (No LM2018121) financed by the MEYS and the OP RDE (the CETOCOEN EXCELLENCE project No. CZ.02.1.01/0.0/0.0/17_043/0009632) for supportive background. This work was supported by the European Union's Horizon 2020 research and innovation program under grant agreement No. 857560. This publication reflects only the author's view, and the European Commission is not responsible for any use that may be made of the information it contains. The Czech‐Norwegian collaboration was supported by the projects EHP‐BFNU‐OVNKM‐3‐048‐2020 and EHP‐BFNU‐OVNKM‐4‐174‐01‐2022 from EEA/Norway grants 2014–2021, Subprogram: Bilateral Fund.

## CONFLICT OF INTEREST STATEMENT

The authors declare no conflicts of interest.

## Supporting information


Data S1.

## Data Availability

The data that supports the findings of this study are available in the supplementary material of this article.
